# A case report of COVID-19 in refractory myasthenia: Outcome with remdesivir and dexamethasone

**DOI:** 10.1097/MD.0000000000025701

**Published:** 2021-05-07

**Authors:** Sachin M. Bhagavan, Swathi Beladakere Ramaswamy, Raghav Govindarajan

**Affiliations:** Department of Neurology, University of Missouri Health Care, Missouri.

**Keywords:** coronavirus disease 2019, dexamethasone, myasthenia gravis, remdesivir, steroids

## Abstract

**Rationale:**

Myasthenia gravis (MG) patients are at increased risk of COVID-19 infection and its complications due to chronic immunosuppression. COVID-19 infection can also increase the risk of myasthenia exacerbation.

**Patient concerns:**

The patient presented with respiratory distress, fever and chills and was diagnosed with COVID-19 pneumonia. His past medical history includes seropositive generalized MG diagnosed in 2019, hypertension, atrial fibrillation and congestive heart failure with reduced ejection failure.

**Diagnoses:**

Refractory seropositive generalized MG having COVID-19 pneumonia and respiratory failure (needing mechanical ventilation) with sepsis.

**Intervention:**

Use of intravenous remdesivir and dexamethasone and patient's myasthenic exacerbation (due to COVID-19 and its complications) was successfully treated with plasmapheresis.

**Outcomes:**

Patient was successfully weaned off ventilator to trach collar and was discharged to inpatient rehabiliation. He was followed up 1 month post hospital discharge and was on trach collar.

**Lessons:**

This case report illustrates early use of the combination therapy might be beneficial in refractory myasthenia gravis cases even with chronic immunosuppression and severe COVID-19 infection.

## 1 Introduction

Myasthenia gravis (MG) patients are at increased risk of COVID-19 infection and its complications due to chronic immunosuppression. COVID-19 infection can also increase the risk of myasthenia exacerbation/crisis.^[1]^ Currently remdesivir and dexamethasone have been used for management of severe COVID-19.^[2]^ This case report describes a patient with refractory seropositive generalized MG having severe COVID-19 pneumonia, respiratory failure with sepsis and having a successful outcome with use of intravenous remdesivir and dexamethasone. In addition, patient's myasthenic exacerbation (likely due to COVID-19) was successfully treated with plasmapheresis.

## 2 Case description

A 77-year-old male patient was admitted to medical intensive care unit from outside hospital after experiencing respiratory distress, fever, chills, and diagnosed with COVID-19. He has a history of seropositive (positive acetylcholine receptor modulating antibody) MG diagnosed in 2019. His Myasthenia Gravis Foundation of America Class was IVb at presentation with severe dysphagia, eighty-pound weight loss over 6 months, severe slurred speech and drooling. His course was complicated by recurrent respiratory failure needing tracheostomy and percutaneous gastrostomy tube. At the time of COVID-19 diagnosis, he was on pyridostigmine 60 mg 3 times a day, prednisone 40 mg daily and maintenance plasmapheresis (3 exchanges every 4 weeks). He was also on mycophenolate 1000 mg BID but this had been stopped at the nursing home he was staying due to unknown reasons. His tracheostomy was decannulated and he used non-invasive ventilation at night. His MG activities of daily living score (MGADL) was 12, MG composite (MGC) score was 30.

At presentation he was tachypneic, oxygen saturation 90% on 4l nasal cannula, tachycardic, neck flexion strength 2/5 (medical research council grading), shoulder abduction 2/5, hip flexion 2/5 and near complete ptosis. His MG composite (MGC) at that time was 36.

Labs on admission was significant for leukocytosis, elevated lactic acid, ferritin, procalcitonin, C-reactive protein, D-dimer and lactate dehydrogenase. CK level was normal (77U/L). On day 2 of admission his oxygen requirement increased to 15l, negative inspiratory force was -13 and Forced Vital Capacity was 0.875 L after which he was placed on the pressure control ventilation. He was started on intravenous remdesivir with an initial dose of 200 mg followed by 100 mg daily (for 4 days) and intravenous dexamethasone 6 mg (10 days). His blood culture grew methicillin resistant staphylococcus aureus and he underwent port removal. His echocardiogram showed no vegetation/thrombus. He was started on antibiotics for a total of 21 days.

**Figure 1:**
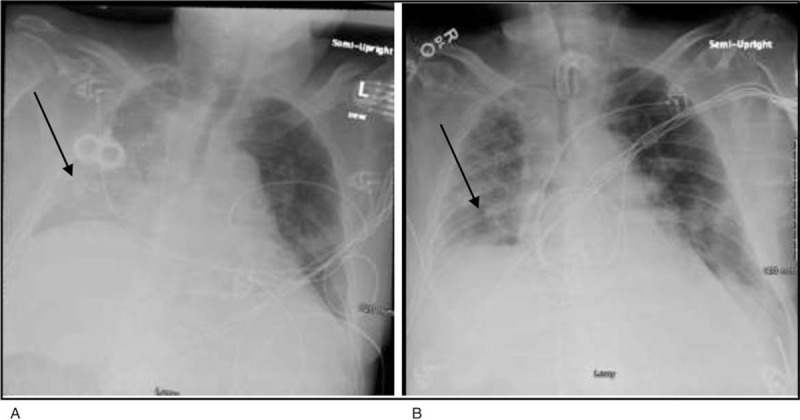
A and Figure 1B (left to right) shows chest x ray of the patient on day 1 of admission and on day 8 of admission respectively.

His chest x-ray anteroposterior view on day 1 showed bilateral air opacities concerning for multifocal infection (Fig. 1A) with improvement seen on day 8 of admission (Fig. 1B). He was tolerating pressure support and was switched to trach collar on day 9.

We suspected MG exacerbation on admission and was started on plasmapheresis (completed 5 sessions, 1 session every other day). His tracheostomy was capped successfully on day 19 of admission and was discharged to inpatient rehabilitation after 21 days of hospital stay. We restarted his pyridostigmine and prednisone. His exam at discharge showed neck flexion strength 3/5, shoulder abduction 3/5, hip flexion 3/5 and improvement in ptosis. He was followed up in neurology clinic at 1 month post discharge and was doing well. It was decided to start eculizumab in the clinic follow up. His MGC at that time was 28.

## 3 Discussion

Current management of severe COVID-19 infection include the use of remdesivir and dexamethasone.^[2]^ Beigel et al found remdesivir group had shorter time to recovery with a median of 10 days as compared to 15 days in placebo with rate ratio for recovery 0.98 (95% CI 0.70–1.36) on mechanical ventilation.^[3]^ However, the study did not stratify patients based on immunosuppressive therapy or analyze patients with MG.^[3]^ Use of dexamethasone for up to 10 days has also shown to be beneficial among hospitalized patients with COVID-19 with lower mortality in patients on invasive mechanical ventilation.^[4]^

Our patient had severe MG with a complicated course requiring tracheostomy and percutaneous endoscopic gastrostomy tube early after the diagnosis and was chronically immunosuppressed. He was living in a nursing home which increased his risk of COVID-19 infection. Use of remdesivir (for 5 days) and dexamethasone (for 10 days) might have prevented worsening respiratory status and also allowing for weaning off the ventilator. Although further studies are needed to evaluate the efficacy of dexamethasone and remdesivir, this case illustrates early use of the combination therapy might be beneficial even in refractory MG with chronic immunosuppression and severe COVID-19 infection.

Severe respiratory failure (i.e., myasthenic crisis) can occur secondary to respiratory muscle weakness in approximately 15% of patients^[5]^ and infections (such as COVID-19) are common triggers. Recent guidelines for management of MG with COVID-19 suggest treatment to be tailored to individual patients and to continue the standard of care for MG.^[5]^ While management of myasthenic crisis with COVID-19 infection do not have a clear guideline.^[6]^ Plasmapheresis seems to be safe and effective in patients with COVID-19 pneumonia and MG exacerbation and might be beneficial due to its rapid onset to treat respiratory compromise.

## 4 Conclusion

Although further studies are needed to evaluate the efficacy of dexamethasone and remdesivir this case illustrates early use of the combination therapy might be beneficial in refractory myasthenia gravis cases even with chronic immunosuppression and severe COVID-19 infection.

## Author contributions

**Conceptualization:** Sachin M Bhagavan, Swathi Beladakere Ramaswamy, Raghav Govindarajan.

**Writing – original draft:** Sachin M Bhagavan.

**Writing – review & editing:** Sachin M Bhagavan, Swathi Beladakere Ramaswamy, Raghav Govindarajan.
